# Integrated analysis of nano Schiff base complex for bioelectronic applications

**DOI:** 10.1038/s41598-025-16628-8

**Published:** 2025-08-25

**Authors:** Emad Mousa, Ahmed K. Tammam, Ahmed M. Refaat, Gehad G. Mohamed

**Affiliations:** 1 Physics Department, Faculty of Science, Cairo University, Giza, Egypt; 2 Chemistry Department, Faculty of Science, Cairo University, Giza, Egypt; 3 Nanoscience Department, Basic and Applied Sciences Institute, Egypt-Japan University of Science and Technology, New Borg El Arab, Alexandria, Egypt

**Keywords:** Schiff base, Ultrasonic studies, Conduction mechanism, Antimicrobial activity, MCF-7 cell line, Biological techniques, Biophysics, Cancer, Materials science, Chemical physics, Condensed-matter physics

## Abstract

Schiff base complexes possess biological activity and electronic features, making them suitable for integration into both core and auxiliary components of bioelectronic technologies. However, integrated studies addressing their electrical, biological, and mechanical properties remain limited. This work investigates a Cu(II) complex based on a Schiff base ligand derived from 2-hydroxy-1-naphthaldehyde and 1,8-diaminonaphthalene. Comprehensive textural analyses using XRD, HRTEM, FESEM, AFM, and N₂ adsorption revealed high surface area, nanoscale morphology, and porosity, which are advantageous for antimicrobial and therapeutic bioelectronic platforms. Mechanical characterization via ultrasonic pulse-echo indicated auxetic behavior, a rare and valuable trait for flexible substrates. The complex also exhibited high ionic conductivity, facilitating charge transport in aqueous environments and contributing to antimicrobial efficacy through ionic disruption of microbial membranes. Thermal analyses showed a phase transition at 44 °C and decomposition onset at 70 °C. A temperature-induced insulator-to-metal transition was observed, suggesting potential for thermally activated sensing, temperature-triggered drug release, and adaptive signal modulation. Biological assays confirmed strong antimicrobial activity, with a 30 mm inhibition zone against Bacillus subtilis (agar well diffusion), and potent cytotoxicity against MCF-7 breast cancer cells, with an IC₅₀ of 18.4 μg/mL (MTT assay). These biological properties enhance the complex’s biocompatibility and support its role in long-term bioelectronic device stability, particularly in applications where infection control is essential.

## Introduction

Schiff bases and their metal complexes are extensively employed chemical compounds known for a broad spectrum of biological activities, including antioxidant and anti-inflammatory^1^, antibacterial and antifungal^2^, antiviral^3^, as well as anticancer and antiproliferative characteristics^4,5^. Beyond their biological significance, these compounds find extensive applications as corrosion inhibitors^6^, pigments^7^, electrochemical sensors^8^, smart precursor for the synthesis of nano oxides^9^, and polymer stabilizers^10^. Moreover, their potential as photosensitizers in dye-sensitized solar cells (DSSCs) has been demonstrated in several studies^11,12^.

The importance of Schiff base ligands stems from their ability to coordinate with metal ions, forming complex structures that can be precisely engineered without undergoing irreversible transformations^13^. This coordination is crucial because it enables scientists to tailor the entire properties of the resulting compounds for a variety of applications^14,15^. By adjusting the metal center and the Schiff base ligand, researchers can fine-tune aspects like conductivity and band gap energy, which are vital for the performance of electronic and optoelectronic devices^11^.

Bioelectronics is an emerging interdisciplinary field that aims to establish a synergy between electronics and biology^16^. It encompasses the development of biocompatible electronic devices, in vitro and in vivo biosensors, biologically derived materials for electronics and optics, and electronic materials synthesized through biological processes. Additionally, it includes technologies for imaging and interfacing with individual biomolecular functional units^17^. A wide range of epidermal and implantable bioelectronic devices are now in use, including glucose sensors, cardiac pacemakers, and electrocorticography systems^18^.

Microbial adhesion and biofilm formation on implantable or wearable bioelectronic devices can significantly increase impedance, degrade signal fidelity, and trigger inflammatory responses. This underscores the importance of developing materials that integrate electronic functionality with built-in infection control, particularly when applied as protective coatings on bioelectronic implants to safeguard against microbial contamination and oxidative degradation^19,20^.

The antimicrobial and electronic multifunctionality of Schiff base metal complexes has been confirmed in several studies. For instance, Nassir et al. explored the multifunctionality of some nano metal complexes based on Schiff base ligands derived from 2-hydroxy-1-naphthaldehyde and antiviral valacyclovir. The Cu(II) complex of the synthesized ligand showed semiconducting nature in addition to its antitumor activities^21^. More of that, Mohanan et al. synthesized a Schiff base ligand that is derived from 2-hydroxy-1-naphthaldehyde and 2-amino-3-carboxyethyl-4,5-dimethylthiophene. The metal complexes based on this ligand demonstrated semiconducting nature in addition to their antimicrobial activities^22^.

While Schiff base complexes are widely recognized for their chemical biological, and electronic versatility, understanding their mechanical properties is essential for advancing their use in flexible electronics, implantable systems, and smart bioelectronic coatings, where durability and biocompatibility are paramount^23^. Yet, there is a lack of integrated research on Schiff base complexes that concurrently examines their electrical properties, biological efficacy, and mechanical integrity.

A notable Schiff base ligand, synthesized through the condensation reaction of 2-hydroxy-1-naphthaldehyde and 1,8-diaminonaphthalene, has demonstrated antimicrobial activities when complexed with certain metals^24,25^. To the best of our knowledge, the AC conductivity and mechanical properties of this complex has not yet been investigated.

In this work, the AC conductivity of Cu(II) complex based on a Schiff base ligand derived from 2-hydroxy-1-naphthaldehyde and 1,8-diaminonapthalene is studied across various temperature and frequency ranges. Thermal analyses, including thermogravimetric analysis (TGA) and differential scanning calorimetry (DSC), are employed to further elucidate the structural changes of the complex within the studied temperature range. Additionally, the ultrasonic pulse-echo technique is utilized to explore the mechanical properties of the complex by determining the acoustic attenuation coefficient, acoustic impedance, and other rigidity parameters. Moreover, textural parameters such as crystallinity, particle size, size distribution, surface area, and surface morphology are analyzed using X-ray diffraction (XRD), high resolution transmission electron microscope (HRTEM), field emission scanning electron microscope (FESEM), atomic force microscope (AFM), and N_2_ gas adsorption techniques. Finally, the Schiff base ligand and its Cu(II) complex are evaluated for their in vitro biological activity using the agar well diffusion method against six microbial strains as well as anticancer activity against MCF-7 cell line using MTT assay.

## Experimental

### Materials and reagents

Analytical reagent grade (AR) chemicals with the finest purity attainable were utilized. The chemicals used include 1,8-diaminonapthalene, 2-hydroxy-1-naphthaldehyde (Sigma-Aldrich) and CuCl_2_.2H_2_O (BDH). Ethyl alcohol (95%), N,N-dimethylformamide (DMF), and diethyl ether were used as solvents.

### Synthesis of the Schiff base ligand and Cu(II) complex

The present ligand and its Cu(II) complex were synthesized as reported in literature^24,26,27^. In brief, Schiff base ligand was firstly synthesized by the condensation of 1,8-diaminonapthelene (25.00 mmol, 4 g) dissolved in hot ethanol (60 °C) and 2-hydroxy-1-naphthaldehyde (25.00 mmol, 8.6g) dissolved in hot ethanol (70 °C) in 1:2 molar ratio. The mixture was left 3 h for reaction under reflux. The violet obtained yield was filtrated, washed with ethanol repeatedly and then dried under vacuum over anhydrous CaCl_2_.

The Cu(II) chelate was synthesized by mixing the prepared Schiff base ligand (2 mmol, 0.5 g) which is dissolved in DMF (20 ml; 60 °C) with the Cu(II) ion (2 mmol, 0.34 g CuCl_2_.2H_2_O) which is dissolved in absolute ethanol (20 ml; 60 °C). The mixtures obtained were subjected to reflux for a duration of 1 h, to precipitate the complex. The precipitate was filtrated followed by multiple washing with diethyl ether. It was then purified by recrystallization using hot ethanol and subsequently dried under vacuum conditions with anhydrous calcium chloride.

**Fig. 1:**
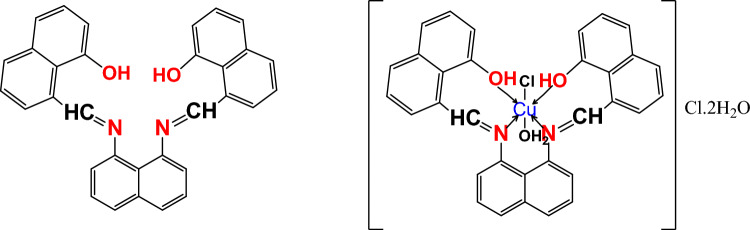
Structure of Schiff base ligand and its Cu(II) complex.

To confirm successful preparation, the compounds were characterized using elemental analyses (supplementary material), FT-IR (Supplementary Fig. 1), ^1^H-NMR (Supplementary Fig. 2), mass spectra (Supplementary Fig. 3), molar conductivity (supplementary material), and UV–Vis analysis (Supplementary Fig. 4). The results confirmed the successful synthesis of the Cu(II) complex with a 1:1 metal-to-ligand stoichiometric ratio. The structures of the ligand and its Cu(II) complex are illustrated in Fig. 1.

### Instrumentation

The crystallinity of the synthesized complex was analyzed using an X-ray diffractometer (XRD; Empyrean, Malvern Panalytical Company). The measurements were conducted at room temperature using Cu-target Kα radiation (0.154 nm) at a scanning rate of 1 degree per minute, within a (2θ) range; 5 to 90 degrees. Transmission electron microscope (TEM) (model EM-2100, High-Resolution) at voltage 200 kV and field emission scanning electron microscope (FE—SEM; Quanta 250 Field Emission Gun) were used to analyze the size and morphology of the complex. In addition, roughness profile was carried out by an atomic force microscope (AFM 5600 LS Agilent Technology Company). Sample preparation for AFM measurement involved ultrasonication of powdered material in deionized water for 45 min and the resulted suspension was deposited onto a mica slide. The measurement conditions were set to a size of 100 × 100 nm, a speed of 0.4 in./sec, and an I Gain of 0.4 and P Gain of 18, utilizing contact mode. N_2_ adsorption at 77 K using a pore size analyzer (NOVAtouch series, Quantachrome Instruments) is used to assess the surface area and pore size distribution of the complex. Vacuum outgassing of the sample was processed at 40 °C for 24 h before measurements.

The pulse echo technique was used to perform the ultrasonic studies^28^. The system consists of receiver/transmitter transducers (S12Y2 and S12HB2, Karl Deutsch), an ultrasonic flaw detector (USN60, GE inspection technologies), an oscilloscope (LeCroy W, wave Jet 354 A), and reference standard blocks (VI and VII). Ultrasonic studies were performed according to ASTM E114-15 standard at normal climate (temperature = 21 °C ± 2 °C and humidity = 52% ± 5%)^29^. The density was determined experimentally using an immersion method based on Archimedes’ principle.

A Shimadzu TG‐50H thermal analyzer was used to carry out thermogravimetric analysis (TGA) of the complex from room temperature to 1000 °C at a heating rate of 10 °C/min under nitrogen atmosphere. Differential scanning calorimetric analysis (DSC) was performed using a DSC‐60 Shimadzu analyzer in the temperature range 25 to 120 °C at a rate of 10 °C/min under nitrogen atmosphere.

AC electrical conductivity analysis was conducted using a computer controlled LCR bridge (HIOKI, 3532–50 LCR HiTESTER, JAPAN) in the frequency range 50 Hz – 5 MHz. The measurements were taken in the temperature range (303 K – 393 K) using a home‐built, computer‐controlled electric oven (± 0.1 °C). Powdered complex was compressed at 25 bar for 2 min and then coated with silver paste. The sample chamber underwent a 12-h process of evacuation in order to completely eliminate any remnants of the silver-paste solvent.

### Biological activity

Agar well diffusion method is utilized to deduce the antimicrobial activity of samples. The method was followed as described in the supplementary material. Zones of inhibition were determined on mm scales and the experiment was replicated three times^30^.

### Anticancer activity

The protocol for assessing anticancer activity was carried out according to the guidelines provided in the supplementary material^31^. The treated sample and the cell control without the investigated compounds were compared. Every experiment was run in triplicate. For the compounds under investigation, the cytotoxic effect on cells was computed^32,33^.

## Results and discussion

### Textural properties

Parameters such as crystallinity, particle size, size distribution, specific surface area, and surface morphology significantly influence the physicochemical properties and functional performance of Schiff base complexes, thereby affecting their suitability for diverse applications. These parameters are considered in this section using XRD, HRTEM, FESEM, AFM, and N_2_ gas adsorption techniques.

### X-ray powder diffraction

**Fig. 2:**
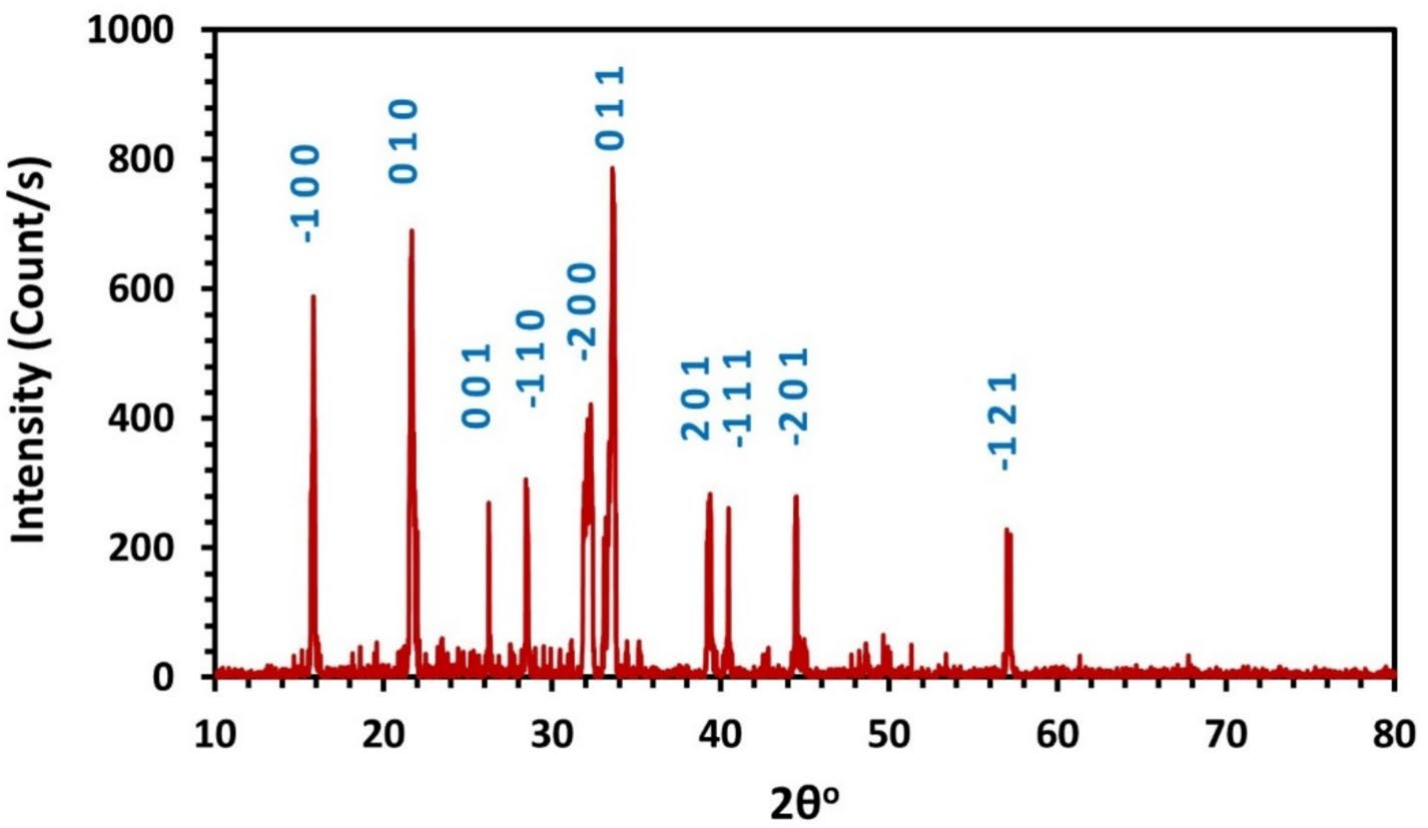
XRD diffractogram of Cu(II) complex.

The X-ray powder diffractogram of Cu(II) complex at room temperature is shown in Fig. (2). CHEKCELL program is used to index the observed diffraction peaks and analyze the obtained data^34^. The complex exhibited triclinic structure, a space group P1, and a cell volume of 78.67 Å ^3^. The lattice parameters are a = 5.6710 Å, b = 4.1410 Å, c = 3.3970 Å, α = 86.720°, β = 82.710°, and γ = 81.790°.

Primary analysis of Fig. (2) revealed the crystallinity index (CI), Eq. (1), which indicates quantitatively the percentage of crystalline and amorphous regions in the synthesized complex.

1 $$CI = \frac{\text{Area of crystalline peaks}}{\text{Area of crystalline and amorphous peaks}} \times 100$$

The synthesized complex exhibits a moderate crystallinity index of 66.9%. In its highly crystalline domains, extended lattices not only facilitate delocalized charge transport, enhancing electronic mobility, but also impart greater hardness and tensile strength while reducing compressibility. Grain boundaries and lattice defects in polycrystalline regions disrupt carrier flow, raise resistivity, and act as stress concentrators that increase stiffness. Amorphous regions introduce localized electronic states that trap carriers and lower conductivity, yet their higher free energy promotes faster dissolution and solubility, thereby enhancing the bioavailability of the complex.

Moreover, the crystallite size (D) and dislocation density (δ) are evaluated according to Scherrer formula:

2 $$D=\frac{K\lambda }{\beta cos\theta }$$

3 $$\delta =\frac{1}{{D}^{2}}$$

where K: shape factor (used here as 0.89), β: full width at half maximum, and θ: Bragg’s angle.

The estimated average crystallite size of the Cu(II) complex is 25.6 nm with dislocation density value of 15.2 × 10^–4^ nm^−2^. Increased dislocation density often leads to work hardening and higher yield strength, but it also scatters charge carriers and phonons, thereby reducing conductivity.

### Field-emission scanning electron microscopy

**Fig. 3:**
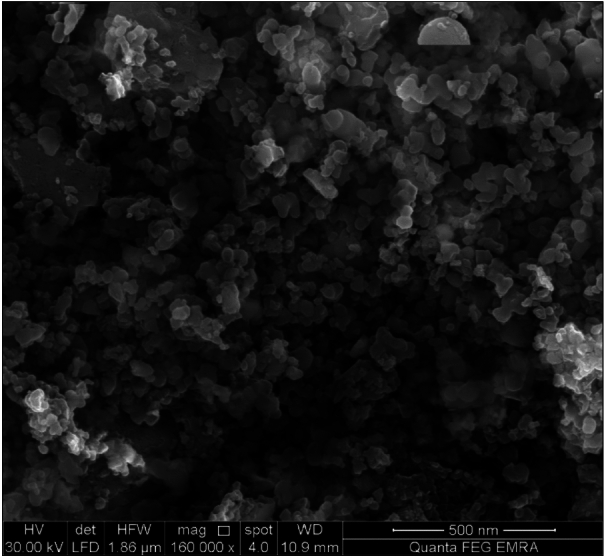
FESEM micrograph of Cu(II) complex.

FESEM micrograph of the complex is illustrated in Fig. (3). A continuous connection between small size grains in non-uniform sizes is observed in the micrograph in addition to some noticeable agglomerations. This morphology is evidence of polycrystalline structure of the sample.

### High-resolution transmission electron microscopy

**Fig. 4:**
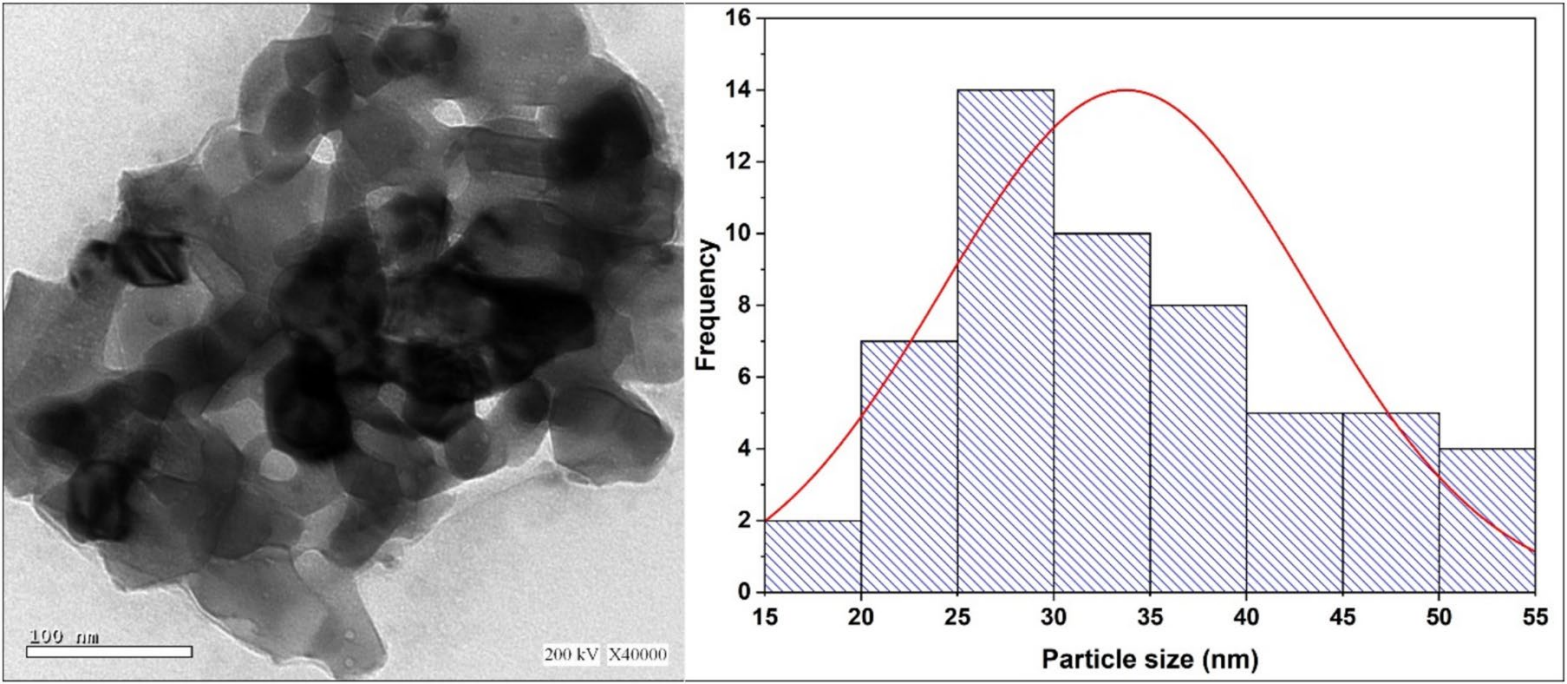
HRTEM micrograph of Cu(II) complex and the corresponding histogram of particle size distribution.

Fig. (4) represented the HRTEM image of synthesized complex and the histogram of particle size distribution based on several snapshots. An average particle size of 33.7 nm is estimated by applying a Gaussian fitting to the histogram. The crystallite size detected by XRD is normally smaller than particle size elucidated by HRTEM due to amorphous content. It is worth mentioning that nano-sized particles dramatically increase surface area to volume ratio, accelerating dissolution rates, delivery and cellular uptake of bioactive ingredients^35^.

### Atomic force microscopy

**Fig. 5:**
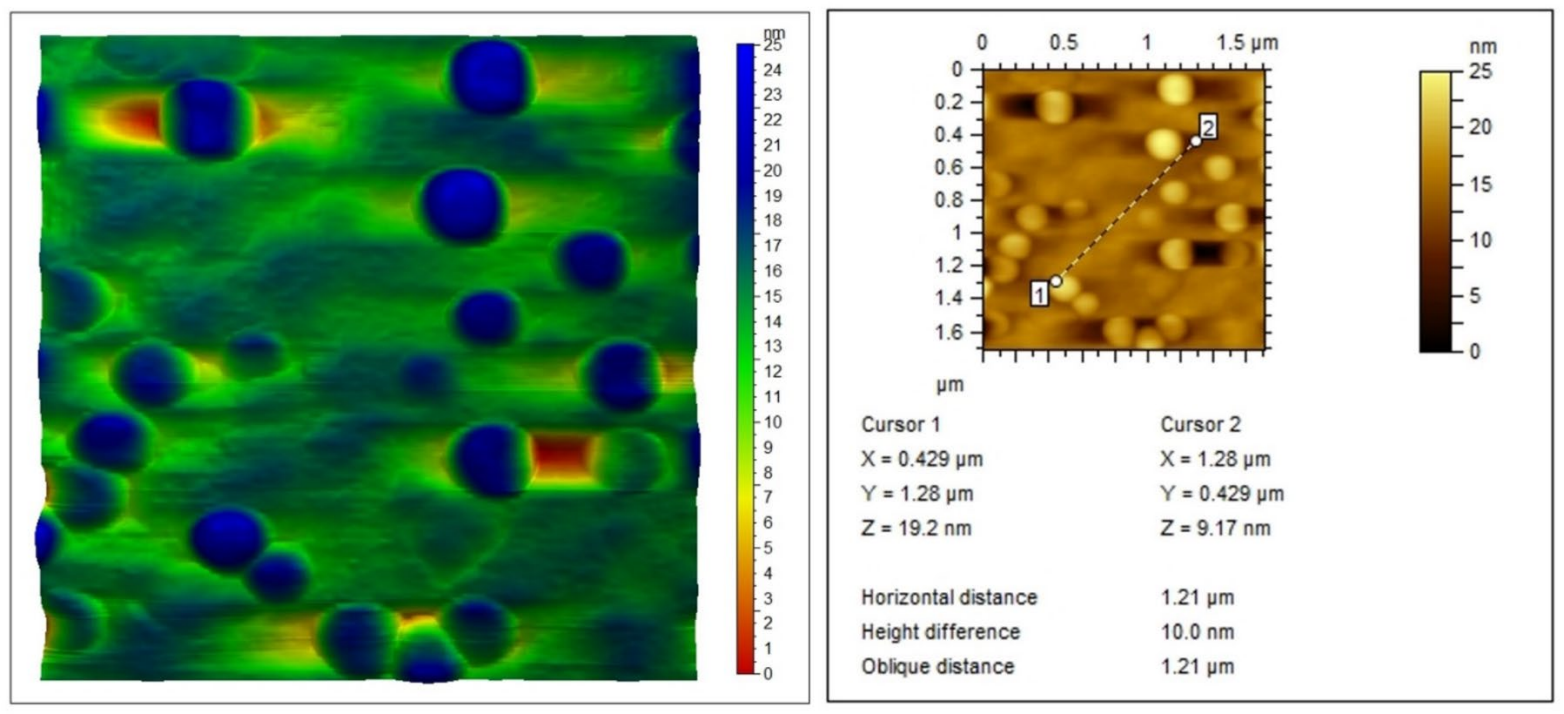
AFM micrograph of Cu(II) complex.

The surface roughness of the investigated nano complex is depicted in Fig. (5). The analysis of the figure revealed the roughness profile of the sample including maximum height (R_z_), maximum peak height (R_p_), maximum valley depth (R_v_), arithmetic mean height (R_a_), skewness (R_sk_), and kurtosis (R_ku_). A high kurtosis value (7.50) indicated that the complex surface consists of spikes. The roughness parameters of the chosen area are summarized in Table (1). Nanoscale valleys and peaks can generate localized electric fields that promote reactive oxygen species generation, enhancing antimicrobial or anticancer activity of the complex^35^.

**Table 1:** Roughness parameters.

R_z_ (nm)	R_p_ (nm)	R_v_ (nm)	R_a_ (nm)	R_sk_	R_ku_
24.7	19.4	5.33	2.70	2.15	7.50

### Adsorption measurements

**Fig. 6:**
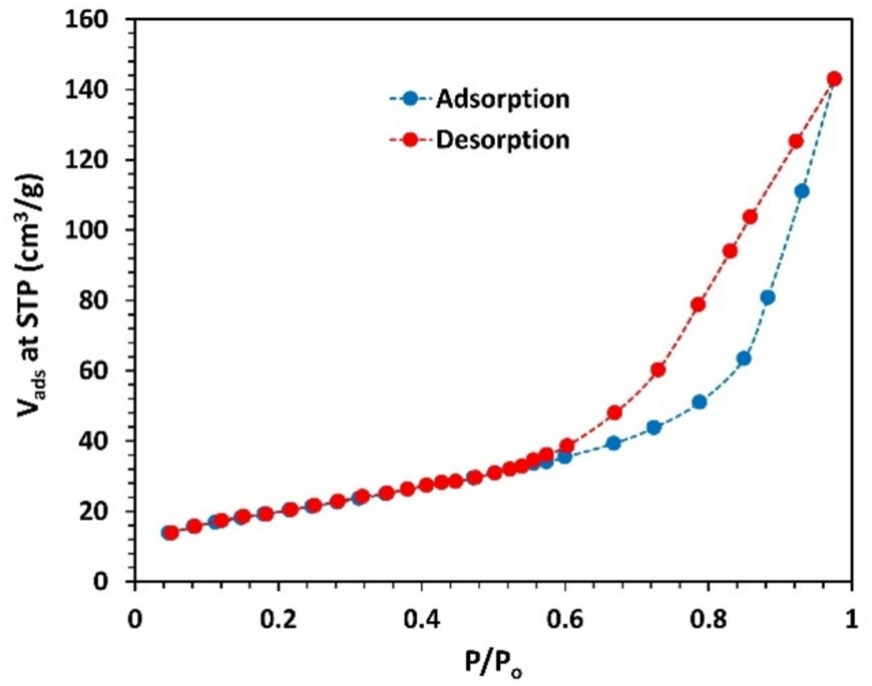
N_2_ adsorption–desorption isotherm.

The N_2_ adsorption–desorption isotherm of the Cu(II) complex, illustrated in Fig. (6), shows a typical type II isotherm with a clear H3 hysteresis loop, which is a common behavior of montmorillonite clays and aggregates of other platy particles^36,37^.

Herein, the surface area of the particles is determined using the Brunauer–Emmett–Teller (BET) equation:

4 $$\frac{1}{W\left[\left({P}_{o}/P\right)-1\right]}= \frac{1}{{W}_{m}C}+ \frac{C-1}{{W}_{m}C}\left(\frac{P}{{P}_{o}}\right)$$

where W: weight of adsorbed gas at given relative pressure $\left(P/{P}_{o}\right)$, W_m_: weight of monolayer of adsorbate, and C: constant.

**Fig. 7:**
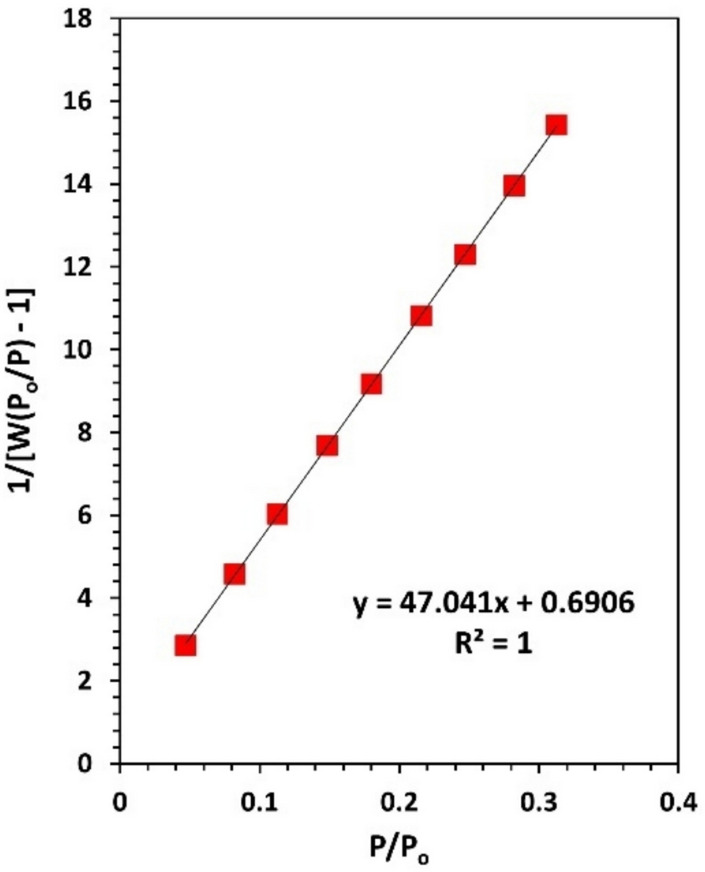
Multipoint (BET) plot of N_2_ adsorption–desorption isotherm for the complex. Solid line is fitting.

Fig. (7) presents a plot of [$\frac{1}{W\left[\left({P}_{o}/P\right)-1\right]}$] vs $\left(\frac{P}{{P}_{o}}\right)$, in the relative pressure range 0.1 ~ 0.31. A linear fitting is applied to the figure in order to calculate C and W_m_. Therefore, the specific surface area of the complex ${SA}_{BET}$ is deduced as:

5 $${SA}_{BET}= \frac{\left({W}_{m}/M\right)N{A}_{cs}}{m}$$

where N: Avogadro’s number, A_cs_: molecular cross section of the adsorbate molecule (0.162 nm^2^ for N_2_ at 77 K), M: molar mass of the adsorbate gas, and m: mass of the sample.

Dubinin-Astakhov (DA) method is used to calculate the pore volume, pore size distribution, and adsorption energy (E) as given by Eqs. (6) and (7)^38^

6 $$V= {V}_{p} exp\left[-{\left(\frac{RT}{E}\right)}^{n}{ln}^{n}\left({P}_{o}/P\right)\right]$$

7 $$\frac{d(V/{V}_{P})}{dr}=3n {\left(\frac{K}{E}\right)}^{n} {r}^{-(3n+1)}\mathit{exp}\left[- {\left(\frac{K}{E}\right)}^{n} {r}^{-3n}\right]$$

where V: pore volume at relative pressure $\left(P/{P}_{o}\right)$, V_p_: whole micropore volume, R: universal gas constant, T: temperature (77 K), n: non-integer value (normally 1 ~ 3), r: pore radius, and K: interaction constant (2.96 kJ.nm^3^/mol for N_2_).

**Fig. 8:**
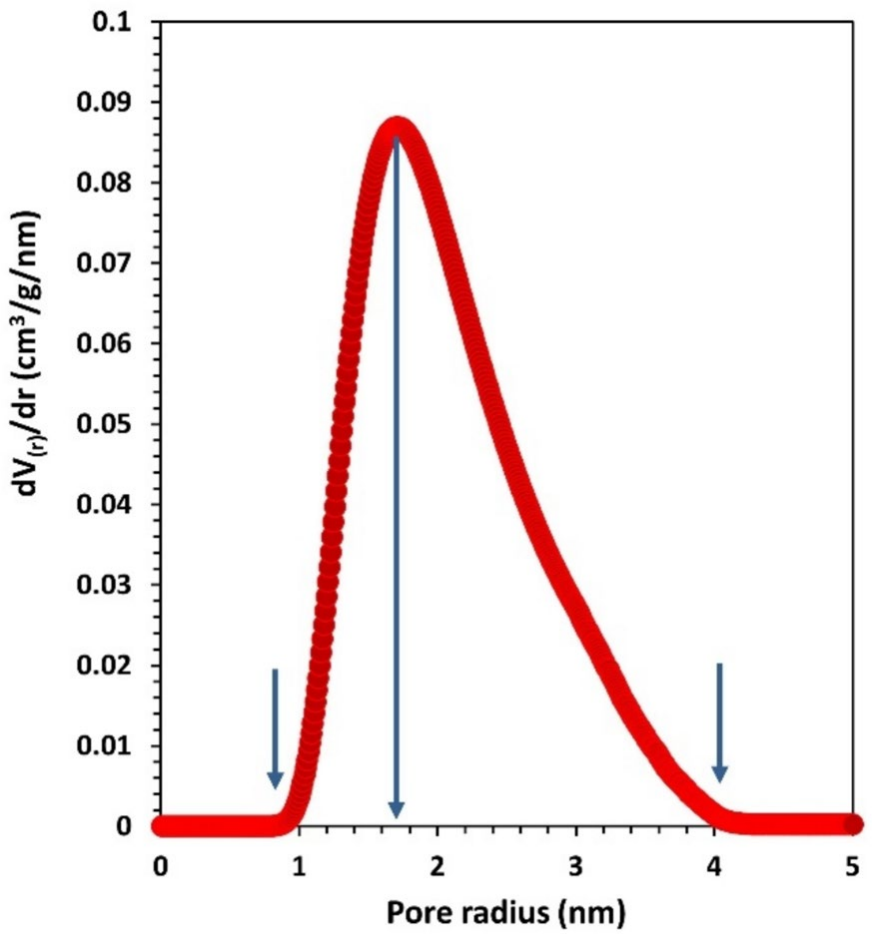
Pore size distribution based on DA model.

Fig. (8) is plotted, based on the analysis of Eq. (7), to demonstrate the heterogeneous distribution of pore size in the micropore–mesopore range (0.9 nm—4.0 nm) within the complex. When Schiff bases serve as carriers or form porous matrices, the distribution of pore sizes controls how much drug can be loaded and how quickly it is released. The heterogeneous pore size distribution in the present Cu(II) complex supports both immediate and prolonged delivery phases.

The specific surface area, pore volume, adsorption energy, and other fitting parameters of Eqs. (4) and (6) are gathered in Table (2). Based on its adsorption energy value, the process can be classified as physisorption.

**Table 2:** BET and DA fitting parameters.

BET	C	69.117
	**SA**_**BET**_** (m**^**2**^**/g)**	72.96
DA	**V**_**pore**_** (cm**^**3**^**/g)**	0.141
	**n**	1
	**r (nm)**	1.710
	**E (kJ/mol)**	0.443

### Ultrasonic measurements

Assessing the mechanical properties of drug formulations and delivery systems is crucial for optimizing both their performance and manufacturing processes^23^. Elastic properties of powder samples can be evaluated using the acoustic ultrasonic technique, a real-time and non-destructive method^39^.

The ultrasonic pulse echo method utilizes high frequency sound waves that penetrate the specimen and then bounce back at the interfaces. The sound waves experience energy damping (attenuation) due to medium defects such as; cracks, grain boundaries, pores, delaminations, and disbonds^40^. Thus, the sound attenuation coefficient (α) is a measure of such energy loss.

8 $$\alpha =\frac{1}{d}\text{ln}(\frac{{A}_{o}}{A})$$

where d: acoustic path traveled by the beam, and A_o_ and A: amplitudes of the beam before and after attenuation.

Another important parameter that can be deduced by ultrasonic pulse echo technique is the acoustic impedance (Z) which refers to the degree of resistance that a medium offers to the movement of sound waves^29^. The acoustic impedance is related to the density (ρ) of material and the speed of the sound waves traveling in the medium (V) as follows:

9 $$Z=\rho V$$

10 $$V = \frac{2x}{t}$$

where x: sample’s thickness, and t: time traveled by the echo in the specimen.

Measurements revealed that α and Z values of Cu(II) complex were 1.1 dB/cm and 13.4 MRayl, respectively.

Hence, the rigidity of the material is characterized by means of longitudinal (V_L_) and shear (V_S_) ultrasonic velocities^41^. Mechanical parameters such as; longitudinal modulus (L), shear modulus (S), bulk modulus (B), Poisson’s ratio (P), Young’s modulus (Y), and microhardness (H) are given by:

11 $$L=\rho {V}_{L}^{2}$$

12 $$S=\rho {V}_{S}^{2}$$

13 $$B=L-\frac{4}{3}S$$

14 $$P=\frac{1-2{(\raisebox{1ex}{${V}_{S}$}\!\left/ \!\raisebox{-1ex}{${V}_{L}$}\right.)}^{2}}{2-2{(\raisebox{1ex}{${V}_{S}$}\!\left/ \!\raisebox{-1ex}{${V}_{L}$}\right.)}^{2}}$$

15 $$Y=2\rho {V}_{S}^{2} (1+P)$$

16 $$H=\frac{(1-2P)Y}{6(1+P)}$$

The calculated mechanical parameters of Cu(II) complex are tabulated in Table (3). Negative value of Poisson’s ratio predicts the auxetic nature of the synthesized complex^42^. Auxetic substrates expand laterally under tension, reducing stress concentrations and enhancing mechanical resilience, which makes them well-suited for stretchable electronics^43^. Moreover, auxetic biomaterials mimic the nonlinear mechanical behavior of soft tissues, promoting improved cell adhesion^44^.

**Table 3:** Density, longitudinal and shear ultrasonic velocities, and mechanical parameters of synthesized complex.

ρ (g/cm^3^)	V_L_ (m/s)	V_S_ (m/s)	L (GPa)	S (GPa)	B (GPa)	P	Y (GPa)	H (GPa)
6.35	2115	1511	28.41	14.49	9.07	−0.02	28.37	5.03

Akseli et al.^45^ measured the elastic properties of commercially available monolayer-coated tablets containing 200 mg of ibuprofen, a nonsteroidal anti-inflammatory drug (NSAID), using the ultrasonic pulse-echo technique. These tablets exhibited an acoustic impedance (Z) of 1.5 MRayl and a Young’s modulus (Y) of 2.38 GPa, which are significantly lower than those observed for the present Cu(II) complex. Compared with ibuprofen tablets, the Cu complex’s higher modulus suggests it may exhibit prolonged or more controlled release profiles, an attractive feature for sustained-delivery formulations.

### Thermal analyses (TGA and DSC)

**Fig. 9:**
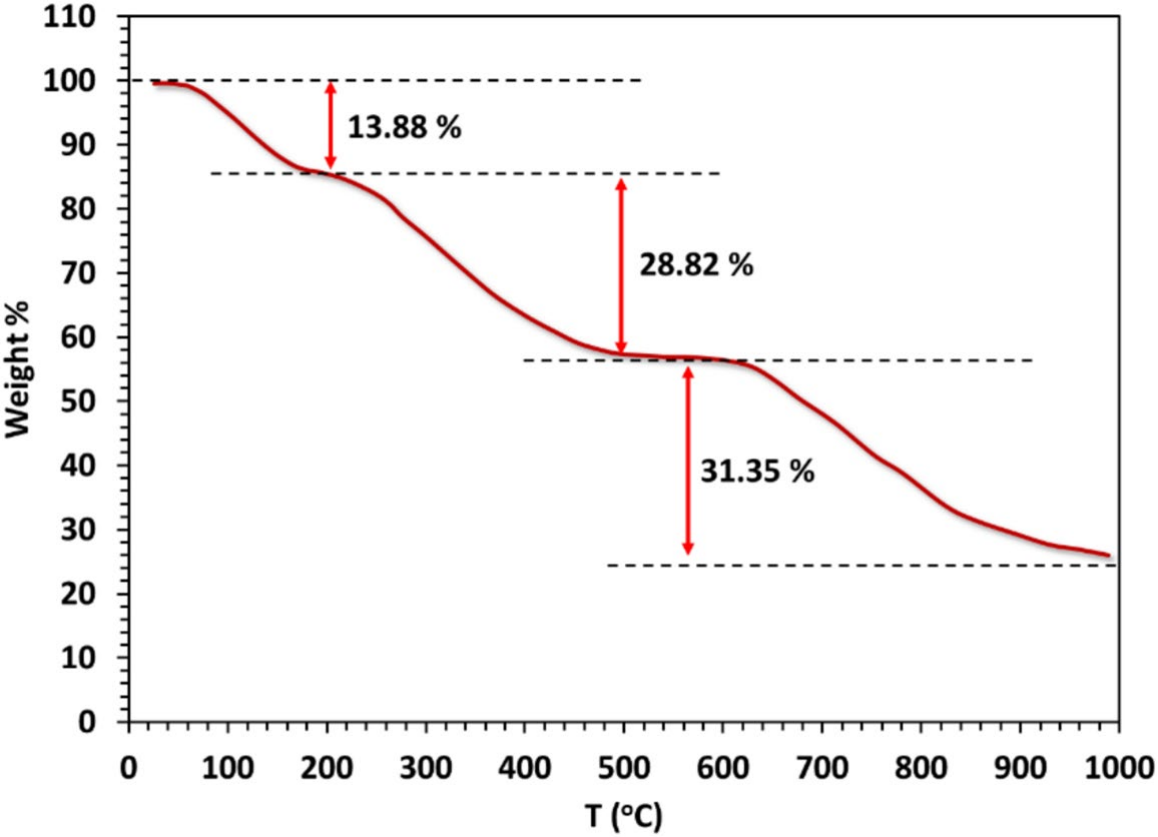
TGA thermogram of the complex.

The thermogravimetric (TGA) curve of the Cu(II) complex is illustrated in Fig. (9). The initial decomposition stage occurs between 70 °C (343 K) to 164 °C (437 K), resulting in a mass loss of 13.88% (calculated mass loss = 13.78%). This mass loss is attributed to the release of half of the chlorine gas molecule (½Cl_2_) and three water molecules (3H_2_O), potentially altering the coordination environment of Cu(II). The second decomposition stage takes place between 249 °C (522 K) and 454 °C (727 K) and can be attributed to the breakdown of another half chlorine gas molecule (½Cl_2_) and C_11_H_18_ molecules, resulting in a measured mass loss of 28.82% (calculated mass loss = 28.48%). The final stage, commencing at 631 °C (904 K) and concluding at 1000 °C (1273 K), results in a mass loss of 31.35% (calculated mass loss = 31.24%), attributed to the elimination of the C_13_H_4_N_2_O molecule. The remaining product of the decomposition process is CuO, contaminated with carbon residues. The total weight reduction is 74.76% (calculated mass reduction = 73.50%).

**Fig. 10:**
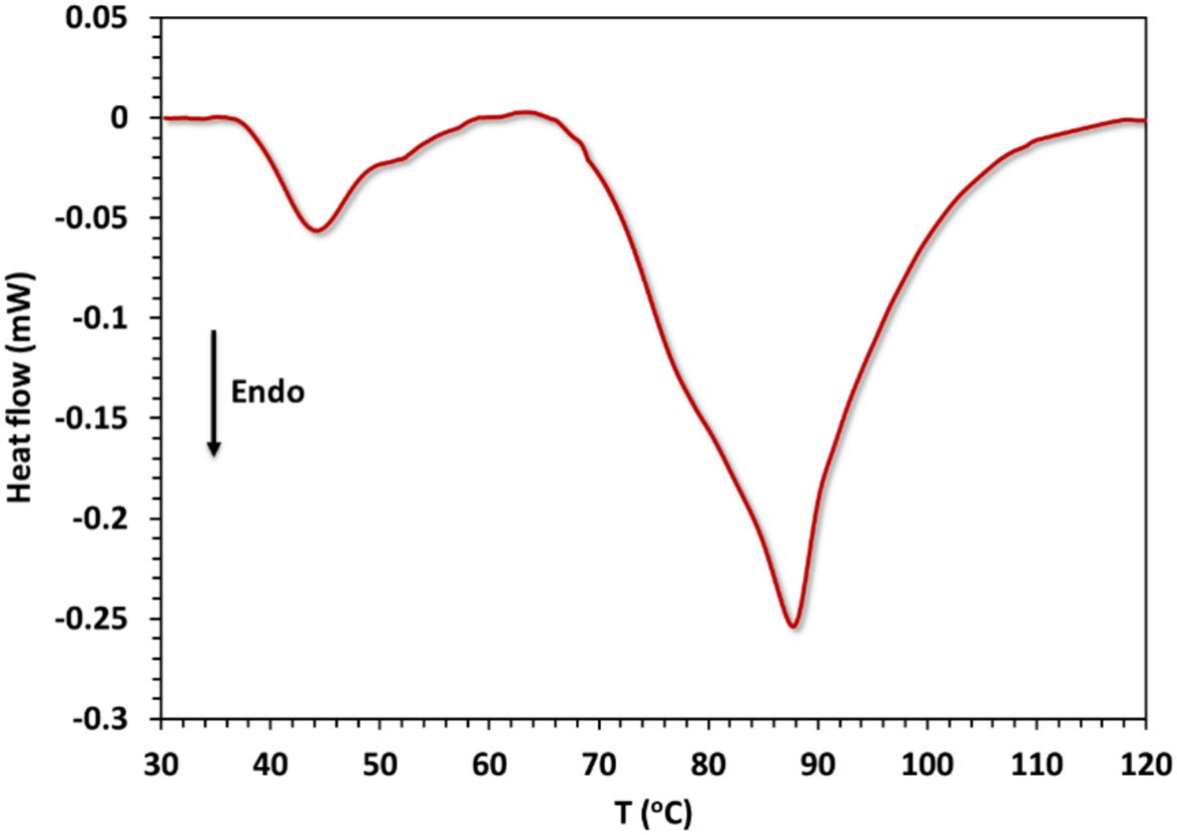
DSC thermograph of the complex.

Two major endothermic peaks appeared in the DSC thermograph of the complex as presented in Fig. (10). The first peak indicates a phase transition occurring between 36.6 °C (309.8 K) and 51.4 °C (324.5 K), with a peak temperature T_pk1_ of 44.1 °C (317.1 K), enthalpy change ΔH of 0.95 kJ/mol, and an entropy change ΔS of 3 J/mol.K. The second peak is broad, appearing in the temperature range of 65.5 °C (338.7 K) to 118.0 °C (391.1 K), with T_pk2_ = 87.5 °C (360.7 K), ΔH = 8.9 kJ/mol, and ΔS = 28 J/mol.K. This broad peak corresponds to the first decomposition step predicted by TGA, with minor differences.

### AC conductivity analysis

AC conductivity analysis is a highly effective tool for exploring the dynamics of charge carriers in both crystalline and amorphous solids^46^.

### Frequency dependence

**Fig. 11:**
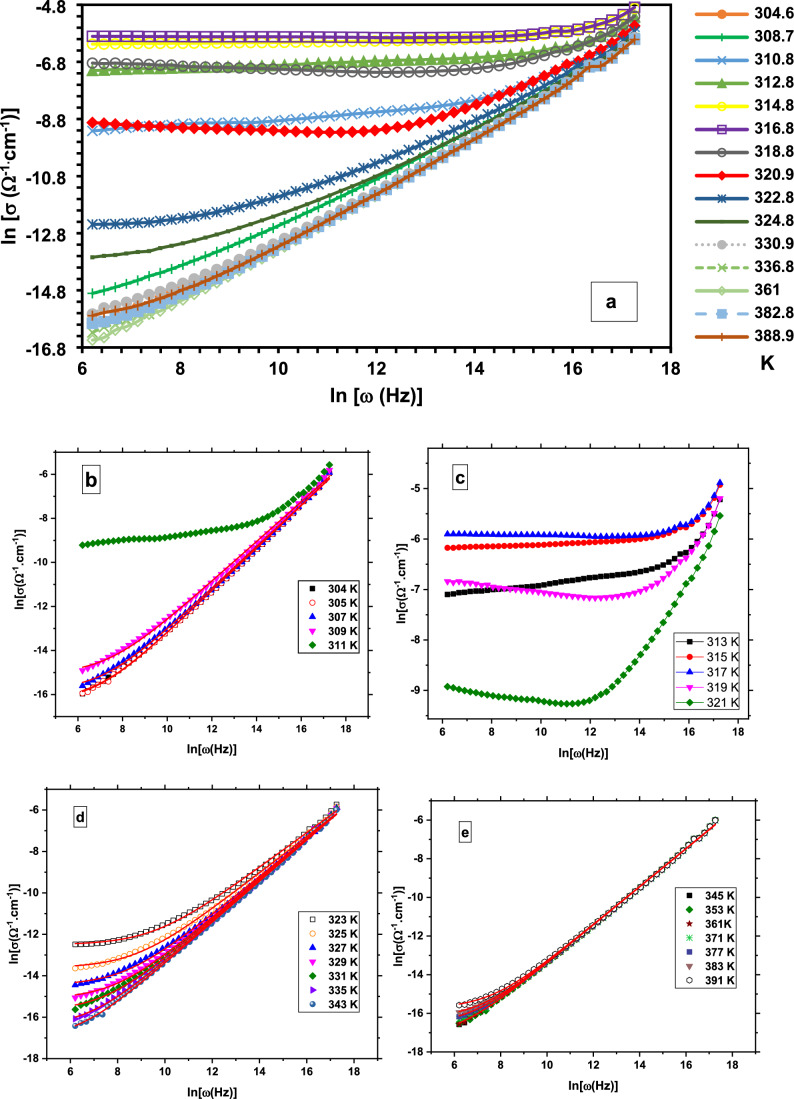
(**a**) [ln (σ)] vs. angular frequency [ln(ω)] at different selected temperatures, (**b**) in temperature range (304- 311) K, (**c**) in temperature range (313- 321) K, (**d**) in temperature range (323- 343) K and (**e**) in temperature range (346- 391) K.

Fig. (11a) shows the variation of [ln σ_ac_)] vs. ln (ω) of Cu(II) complex upon heating. The universal dynamic response (UDR), also known as Jonscher’s power law behavior, is observed in numerous insulators and semiconductors at specific temperatures, resulting in a variation of σ_ac_^47^.

17 $${\sigma }_{ac}={\sigma }_{dc}+A{\omega }^{s}$$

where 0 < s < 1. This behavior is linked to relaxations caused by the movement of electrons or atoms between their equilibrium states, either through hopping or tunneling^48^.

However, varying rates of conductivity increase have been observed across different temperature regions. To clarify this complex behavior, the entire measurement is divided into four distinct phases corresponding to four temperature regions:

a- Phase (I):

Fig. (11b) shows the variation of [ln σ_ac_)] vs. ln (ω) in temperature range (T ≤ 311 K). The conductivity exhibits a consistent increasing trend with rising frequency within this temperature range, with excellent fit to Eq. (17). The conductivity values for this temperature range are consistent with those of insulators.

b- Phase (II):

Fig. (11c) shows the variation of [ln σ_ac_)] vs. ln (ω) in temperature range (313 ≤ T(K) ≤ 321). This temperature range corresponds to a phase transition within the sample as detected by DSC. Below T = 316 K, the conductivity exhibits irregular increasing behavior. At T = 316 K, the conductivity undergoes a drastic change, where σ- value decreases with frequency up to a certain point, after which it starts to increase with increasing frequency. The critical frequency at which the inversion occurs is temperature-dependent, decreasing as the temperature increases. Besides, one can observe that the conductivity value approaches ~ 3 × 10^–3^ Ω^−1^ cm^−1^. This behavior suggests the presence of metallic-like nature in the sample at this temperature range. For all curves in phase (II), the conductivity relations could not be fitted well to Eq. (17).

c- Phase (III):

Fig. (11d) shows the variation of [ln σ_ac_)] vs. ln (ω) in temperature range (323 ≤ T(K) ≤ 343). Differing low conductivity values at low frequencies, with converging high values at high frequencies, have been observed. An excellent fit to Eq. (17) is obtained.

d- Phase (IV):

Fig. (11e) depicts the variation of [ln σ_ac_)] vs. ln (ω) in temperature range (345 ≤ T(K) ≤ 393). This temperature range corresponds to the evolution of half a chlorine gas molecule and water molecules, as identified by TGA and DSC. Within the frequency range of 1.2 kHz to 3.8 MHz, the conductivity exhibits a superimposed linear relationship with temperature-independent behavior.

**Fig. 12:**
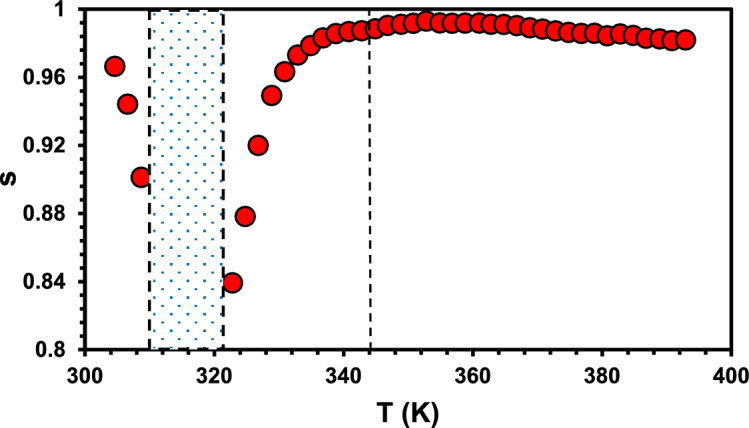
The temperature dependence of the resulting fit parameters (s) of the complex.

However, the behavior of (s) parameter with temperature plays a crucial role in determining the conduction mechanism^49–51^. The fitting parameter (s) has been plotted versus T(K), see Fig. (12). It shows clearly the four phases discussed above. For phase (I) the fitting parameter (s) decreases, (0.97- 0.9), as temperature increases, suggesting quantum mechanical tunneling conduction in this low temperature range. In phase (III), (s) shows increasing behavior with temperature at different rates. Such a behavior suggests small polaron tunneling for phase (III). Correlated barrier hopping is the most probable conduction mechanism for phase (IV) corresponding to high temperature response.

**Fig. 13:**
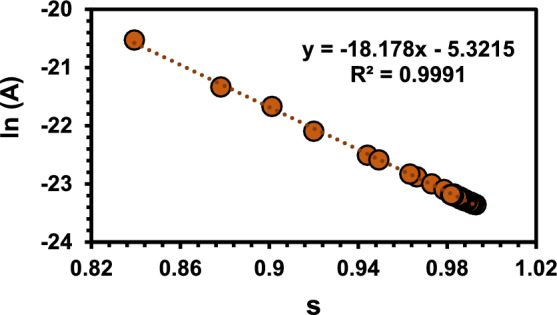
Linear plot of [ln(A)] versus (s) for the complex.

Fig. (13) verified the inverse linear relation between fitting parameters (s) and ln (A) which confirm the fitting accuracy. DC conductivity as obtained from Eq. (17) gave values of ΔE_DC_ = 1.74 eV for phase (I) and ΔE_DC_ = 0.69 eV for phase (IV).

### Temperature dependence

**Fig. 14:**
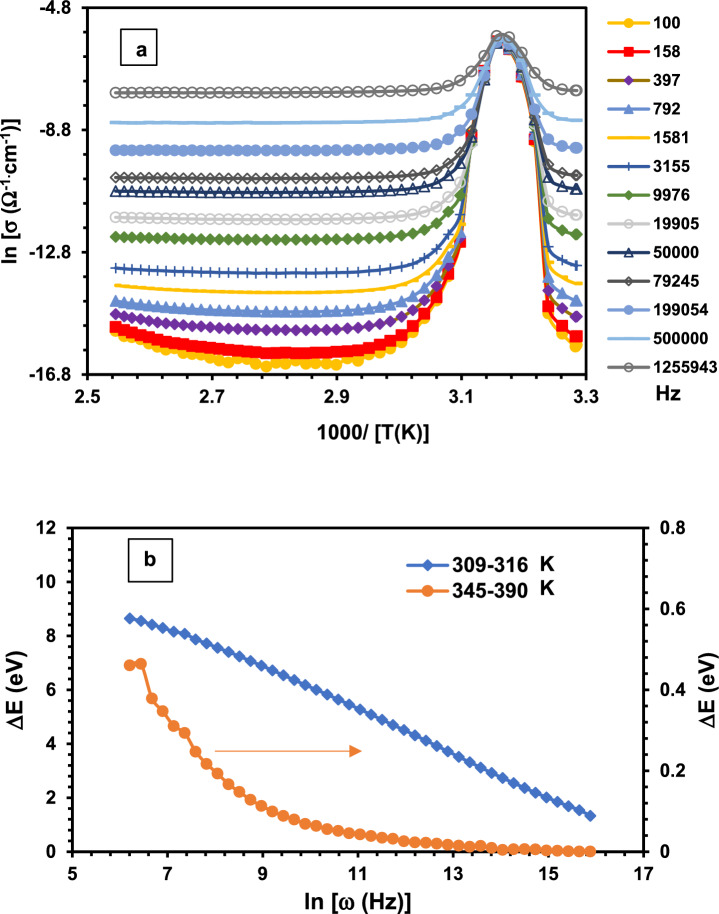
(**a**) [ln (σ)], as a function of reciprocal temperature [1000/T (K)], at selected frequencies and (**b**) Activation energy (ΔE) vs. frequency as ln[f (Hz)] according to Eq. (18).

The variation of [ln σ_ac_)] vs. 1000/T of Cu(II) complex upon heating is illustrated in Fig. (14a). A frequency-dependent relationship has been observed, displaying a wide range of conductivity values from σ = 6 × 10^–8^ Ω^−1^.cm^−1^ at 100 Hz to σ = 6 × 10^–4^ Ω^−1^.cm^−1^ at 1.26 MHz. This range falls within the insulator-semiconductor conductivity spectrum. A clear transition has been observed at T = 316 K, where the conductivity increased at all frequencies and merged with high conductivity value (σ ~ 3.3 × 10^–3 ^Ω^−1^.cm^−1^). The temperature dependence of the AC conductivity (σ_ac_) is thermally activated in two distinct regions (309–316) K and (345–390) K, following the well-known Arrhenius relation:

18 $${\sigma }_{(T)}={A}_{o}\text{exp}(-\frac{\Delta E}{kT})$$

Where ΔE is the activation energy, Ao is the pre-exponential factor, k is Boltzmann constant and T is the temperature.

Fig. (14b) showed the frequency dependence of the calculated activation energies. It showed a decreasing behavior in accordance with the relation^52^:

19 $$\Delta E=\Delta {E}_{o}{\left[1-\text{exp}(-{f}_{o}/f)\right]}^{\alpha }$$

where Δ*E*_o_ is the activation energy in the limit of *f* → 0 and has values 0 < α < 1.

The dependence of ΔE on frequency fits well to Eq. (19) in two temperature regions (309–316) K and (345–390) K. The fitting revealed high activation energy values (6.2 eV – 2.2 eV) in the temperature region (309 K – 316 K), indicating band type conduction. The fitting parameters are E_o_ = 11.125 eV, f_o_ = 1134.5 Hz, and α = 0.179. In contrast, the activation energy values in the temperature region 345 K – 390 K are comparatively small, starting from 0.46 eV and decreasing to almost zero at high frequencies (> 250 kHz). The activation energy values in this region align with proton hopping conduction^53,54^. The fitting parameters are E_o_ = 0.765 eV, f_o_ = 44.19 Hz, and α = 0.554.

The intriguing insulator-to-metal conductivity transition, accompanied by high activation energy values (6.2 eV – 2.2 eV) near 316 K (43 °C), suggests that the complex undergoes a conformational structural phase transition likely involving reorganization of its coordination sphere. Such a transition may planarize the ligand scaffold, thereby enhancing π-electron delocalization throughout the network. However, this thermally induced behavior, indicative of potential for responsive electronic applications, resembles that observed in some organic–inorganic hybrid perovskites^55^.

### Biological activity

#### Antimicrobial activities

The antimicrobial activities of the ligand and its Cu(II) complex were assessed using the agar well diffusion method against six microbial strains. The Schiff base ligand showed biological activity of 14, 12, 10, 11, 10 and 13 mm against *Bacillus Subtilis*, *Streptococcus pneumoniae*, *Escherichia coli*, *Pseudomonas aeruginosa*, *Aspergillus fumigates*, and *Candida albicans* organisms, respectively. In comparison, its Cu(II) complex demonstrated enhanced activity, with inhibition zones of 30, 22, 18, 13, 14 and 20 mm against the same set of organisms, respectively. According to the results, the Cu(II) complex surpassed the Schiff base ligand in its activities against all species of bacteria and fungi. The Cu(II) complex exhibited its highest activity value against *Bacillus Subtilis* (30 mm) and *candida albicans* (20 mm) species. Furthermore, these values were far closer to the ampicillin (32 mm) and amphotericin B (25 mm) standards and greater than the Schiff base ligand (14 mm and 13 mm for *Bacillus Subtilis* and *candida albicans*, respectively).

Therefore, Cu(II) complex is a particularly effective medication against the species *Bacillus Subtilis*. The enhanced antimicrobial activity of the Cu(II) complex arises from multiple synergistic factors, with chelation playing a central role. Chelation reduces the polarity of the metal ion through partial charge sharing with donor atoms and promotes π-electron delocalization across the metal–ligand framework. This increases the complex’s lipophilicity, facilitating its penetration through microbial lipid membranes and enabling efficient attachment and infiltration into the cellular architecture of pathogens^56–59^. Notably, the complex exhibits high molar conductivity (Supplementary material), indicative of its electrolytic nature^60,61^. This property enhances its solubility and ionization in biological media, improving its interaction with microbial cell surfaces and potentially disrupting membrane integrity. The elevated conductivity may also facilitate transport across cell membranes and amplify the compound’s bioavailability at the site of action^62^. In addition, chelation is known to influence key physicochemical parameters such as dipole moment and cell permeability, further contributing to biological efficacy. The Cu(II) ion itself may participate in redox reactions, leading to the generation of reactive oxygen species (ROS) within microbial cells, as supported by previous studies^63^.

The Schiff base ligand previously reported^64^ revealed no antifungal activity against *Aspergillus fumigates*, and *Candida albicans* organisms in contrast to the present Schiff base ligand which showed remarkable activity. Meanwhile, the previously reported Cu(II) complex^64^ showed higher antimicrobial activity than the present Cu(II) complex. The Cu(II) complex reported previously by Abdel-Halim et al*.*^65^ showed higher activity than the Schiff base ligand in accordance to our findings. They reported that the Schiff base ligand had antimicrobial activity values of 15.9, 14.6, 9.8, 10.2, 0 and 12.1 mm while Cu(II) complex had activity of 33.7, 22.6, 15.3, 13.1, 11.5 and 16.5 mm against *Bacillus Subtilis*, *Streptococcus pneumoniae*, *Escherichia coli*, *Pseudomonas aeruginosa*, *Aspergillus fumigates*, and *Candida albicans* organisms, respectively^65^. It was clear from these data that the previously reported Schiff base^65^ had more or less antimicrobial activity comparable with our described Schiff base except it had no activity against *Aspergillus fumigates* in contrast to our results. Similarly, the previously described Cu(II) complex^65^ revealed antibacterial activity close to our reported Cu(II) complex while it had lower antifungal activity in comparison with that reported in this article. This may be attributed to differences in ligand substituents, affecting coordination geometry and lipophilicity. These factors significantly affect how well the complex interacts with fungal cells and, consequently, its effectiveness as an antifungal agent^63^. Moreover, the Cu(II) complex reported by Singh et al*.*^66^ exhibited antibacterial activity against *Escherichia coli* (17.3 mm inhibition zone) and antifungal activity against *Candida albicans* (15.6 mm inhibition zone). These values were lower than those reported for the Cu(II) complex in this article. Thus, the present Cu(II) complex showed a higher antimicrobial and cytotoxic effect, suggesting that the unique structural features of the ligand—particularly the presence of the azomethine, naphthalene ring and phenolic groups play a key role in enhancing the biological activity.

### Anticancer activities

Cancer is regarded as the most serious affliction among numerous human diseases, and unfortunately, there are now no effective treatments or techniques of control accessible for its treatment^67^. The cytotoxicity of the Schiff base ligand and its Cu(II) complex were assessed against breast carcinoma cells (MCF-7 cell line), a prevalent kind of malignancy, using MTT assay. Firstly, the Cu(II) complex was produced and then tested in a single dose experiment on the MCF-7 cell line at a concentration of 100 μg/ml. The IC_50_ (μg/ml), which represents the concentration needed to inhibit 50% of cell viability^68^ was calculated. Based on the findings provided in the experimental section, it is evident that the Cu(II) complex (IC_50_ (μg/mL) 18.4) exhibited significant efficacy for treating breast cancer than the Schiff base ligand (IC_50_ (μg/mL) 25.2). Aazam et al*.*^69^ reported the synthesis of a novel Schiff base ligand and its Cu(II) and Ni(II) complexes. The *in-vitro* cytotoxic activity of the free ligand and its complex compounds was evaluated against two cancer cell lines (HeLa and MCF-7 cells). The Cu complex showed considerable activity against the HeLa and MCF-7 cell lines, as evidenced by the IC_50_ values of 14.04 and 19.25 mM for HeLa and MCF-7 cells, respectively. It was clear that the reported Cu(II) complex had nearly the same IC_50_ values as the previously described Cu(II) complex on MCF-7 cells and higher activity than the one on HeLa cells. It is worth mentioning that, the reported specific surface area of the Cu(II) complex as determined by N_2_ adsorption (33 m^2^/g) was lower than that of the present Cu(II) complex (73 m^2^/g).

## Conclusion

Few studies have concurrently explored the electrical, biological, and mechanical properties of Schiff base complexes. In this study, a Cu(II) complex based on a Schiff base ligand derived from 2-hydroxy-1-naphthaldehyde and 1,8-diaminonaphthalene was synthesized and comprehensively characterized.

Textural analysis revealed a crystallinity of 66.9%, an average particle size of 33.7 nm, nano-scale surface roughness, and a specific surface area of 72.96 m^2^/g, with a heterogeneous pore size distribution. These parameters were determined using XRD, HRTEM, FESEM, AFM, and N₂ adsorption techniques. The combination of high surface area, nanoscale morphology, and porosity suggests rapid dissolution and enhanced solubility, which can facilitate reactive oxygen species (ROS) generation and support prolonged release kinetics, features that are particularly advantageous for bioelectronic platforms involving antimicrobial action or controlled therapeutic delivery. Mechanical properties, evaluated using the ultrasonic pulse-echo method, indicated auxetic behavior, an uncommon and valuable trait for flexible bioelectronic substrates.

Thermal analysis showed a phase transition at 44 °C (DSC) and decomposition onset at 70 °C (TGA). Notably, an insulator-to-metal transition was observed at the transition temperature, enabling temperature-dependent electrical modulation. This thermal responsiveness positions the complex as a candidate for bioelectronic applications such as thermally activated sensing, temperature-triggered drug release, and adaptive signal modulation.

The complex exhibited high ionic conductivity, as evidenced by its molar conductance, supporting efficient charge transport in aqueous environments. This electrolytic behavior likely contributes to its antimicrobial efficacy via ionic disruption of microbial membranes.

Biologically, the Cu(II) complex exhibited superior activity, showing a 30 mm inhibition zone against *Bacillus subtilis* using agar well diffusion method and cytotoxicity against MCF-7 breast cancer cells with an IC₅₀ of 18.4 μg/mL using MTT assay. These results surpass those of the free ligand (14 mm inhibition zone; IC₅₀ = 25.2 μg/mL), indicating a clear enhancement in bioactivity upon complexation. The complex’s biological activity enhances its biocompatibility and contributes to long-term device stability, particularly in bioelectronic applications where infection risk is a major concern. These include skin-contact or implantable devices, wound monitoring platforms, and flexible healthcare electronics requiring integrated sensing and antimicrobial defense.

Overall, these findings highlight the multifunctionality of the Cu(II) Schiff base complex and its potential as a versatile material for bioelectronic devices that require mechanical adaptability, thermal responsiveness, and antimicrobial efficacy.

### Future prospects

Future research should focus on the development of novel Schiff base complexes that seamlessly integrate biological activity with electronic functionality, enabling their use in advanced bioelectronic systems. Equally important is the incorporation of these multifunctional complexes into biocompatible polymers or hydrogels to fabricate flexible, responsive platforms suitable for interfacing with biological tissues. Such hybrid materials hold great promise for applications in wearable diagnostics, implantable sensors, and smart therapeutic devices, where mechanical adaptability, bioactivity, and electronic performance must converge.

## Supplementary Information

Supplementary Information.

## Data Availability

The data that support the findings of this study are available as electronic supplementary materials.
