# Supplementary material for: Integrated analysis of nano Schiff base complex for bioelectronic applications
Source: Sci Rep. 2025 Aug 25;15:31202. doi: 10.1038/s41598-025-16628-8 (PMC12379141; doi:10.1038/s41598-025-16628-8)
Supplement: Supplementary file 1 — Supplementary Information. [file 41598_2025_16628_MOESM1_ESM.docx]

**Integrated Analysis of Nano Schiff Base Complex for Bioelectronic Applications**

Emad Mousa*^1^, Ahmed K. Tammam^1^, Ahmed M. Refaat^2^, Gehad G. Mohamed^2,3^

^1^ Physics Department, Faculty of Science, Cairo University, Giza, Egypt

^2^ Chemistry Department, Faculty of Science, Cairo University, Giza, Egypt

^3^ Nanoscience Department, Basic and Applied Sciences Institute, Egypt-Japan University of Science and Technology, New Borg El Arab, Alexandria, Egypt

* Corresponding Author; e-mail: [emad@sci.cu.edu.eg](mailto:emad@sci.cu.edu.eg)

**Chemical analysis of Schiff base ligand and its Cu(II) complex:**

- **Instrumentation**

Detailed examinations of carbon, hydrogen, and nitrogen were conducted using a CHNS‐932 (LECO) Vario elemental analyzer.

The metals were analyzed by dissolving the solid complex in concentrated HNO_3_ and then dissolving the remaining substance in deionized water. The copper content was determined using inductively coupled plasma atomic absorption spectrometry (ICP-AES).

Mass spectra were recorded by the EI technique at 70 eV using an MS‐5988 GS‐MS Hewlett–Packard instrument

The Fourier transform infrared (FT-IR) spectra were obtained using a PerkinElmer 1650 spectrometer, with a measurement range of 400-4000 cm^-1^, by using KBr pellets.

The ^1^H-NMR spectrum was obtained by recording solutions in DMSO-*d_6_* using a 300 MHz Varian-Oxford Mercury instrument at room temperature. Besides, tetra-methylsilane was used as an internal standard.

A Jenway 4010 conductivity meter was used to measure the molar conductivity (Λ_m_) of the solid complex in DMF (10^‐3^ M solution).

A Shimadzu UVmini‐1240 spectrophotometer was utilized to study the UV–visible spectra.

- **Analysis:**

**Schiff base ligand:**

yield 88%; violet precipitate.

Anal. Calc. for C_32_H_22_N_2_O_2_ (%): C, 82.40; H, 4.72; N, 6.09. Found (%): C, 82.22; H, 4.52; N, 6.00.

Mass (m/z) 468 amu (466 g/mol).

FT-IR (ν, cm^-1^): phenolic OH 3378sh, azomethine (CH=N) 1615sh, phenolic (C-O) 1230m.

^1^H-NMR (300 MHz, DMSO-*d_6_*, δ ppm): 6.37–7.85 (m, 18H, ArH), 9.70 (s, 2H, CH=N) and 10.30 (s, 2H, phenolic-OH).

UV-Vis (λ_max_, nm): 277 (π-π* of phenyl groups), 333 (π-π* of C=N azomethine group) and 413 (charge transfer).

**Cu(II) complex:**

yield 91%; dark brown precipitate.

Anal. Calc. for C_32_H_28_Cl_2_CuN_2_O_5_ (%): C, 58.80; H, 4.28; N, 4.28; Cu, 9.63. Found (%): C, 58.61; H, 4.41; N, 4.22; Cu, 9.75.

Mass (m/z) 655 amu (654.5 g/mol).

FT-IR (ν, cm^-1^): phenolic OH 3435s, azomethine (CH=N) 1629s, phenolic ν(C-O) 1270m, H_2_O stretching of coordinated water 955w and 821s, (M-O) 591w, (M-N) 456w.

Λ_m_ (Ω^-1^mol^-1^cm^2^) = 65. μ_eff_ (BM) 2.03.

UV-Vis (λ_max_, nm): 268 (π-π* of phenyl groups) and 317 (n-π* of C=N azomethine group).

- **Figures:**


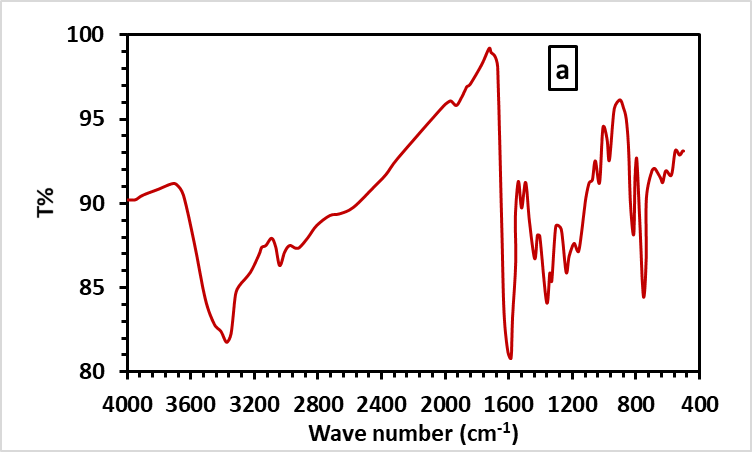


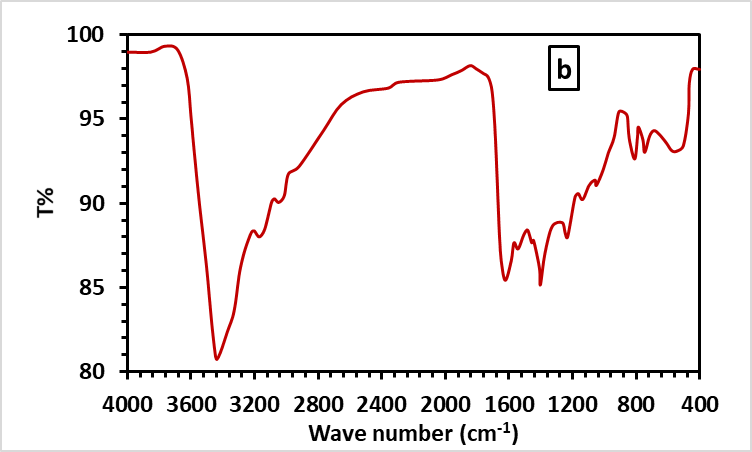


**Supplementary Figure 1.** IR spectra of (a) Schiff base ligand (H_2_L), and (b) Cu(II) complex


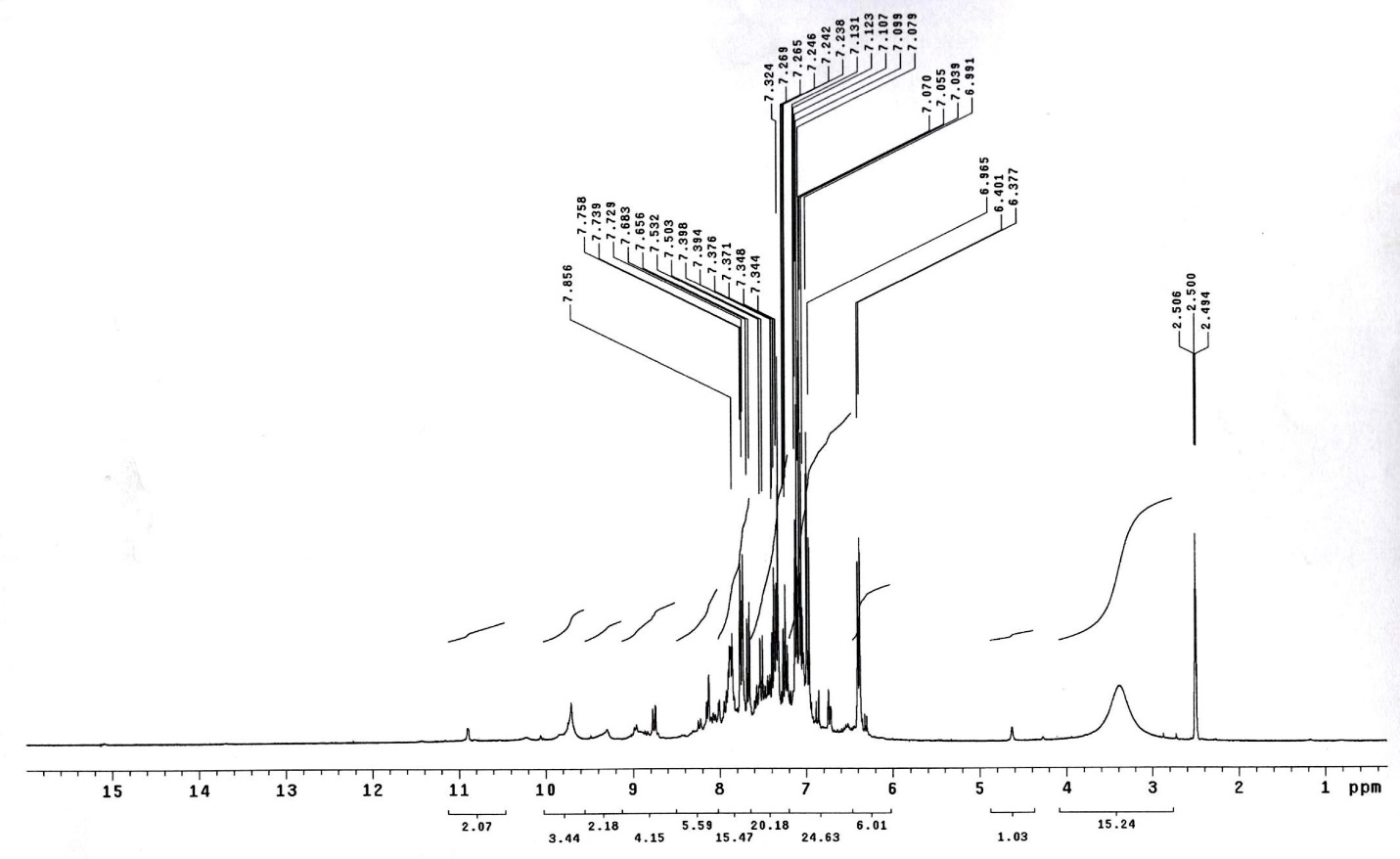


**Supplementary Figure 2.** ^1^H-NMR spectrum of Schiff base ligand


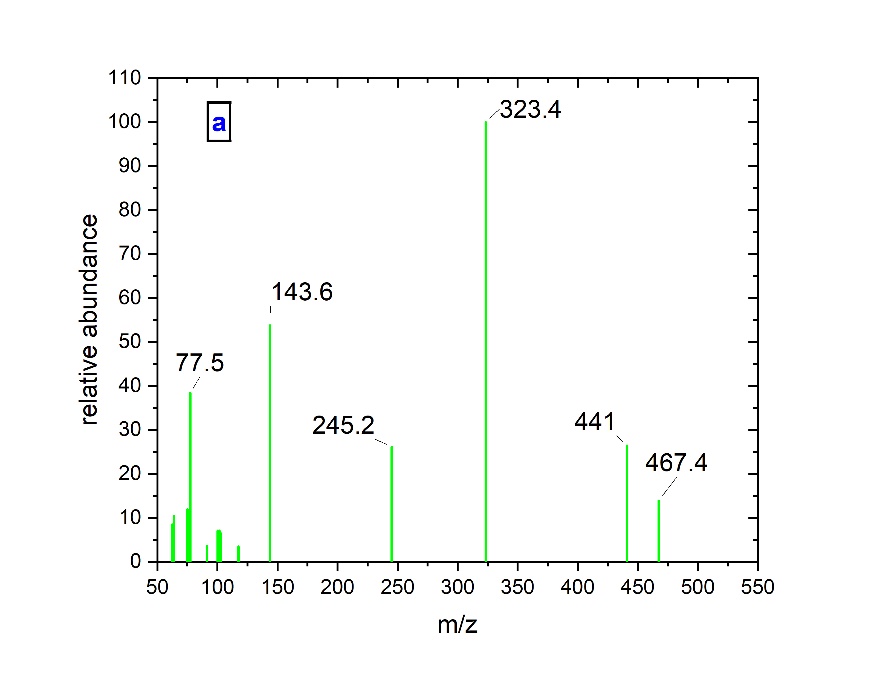


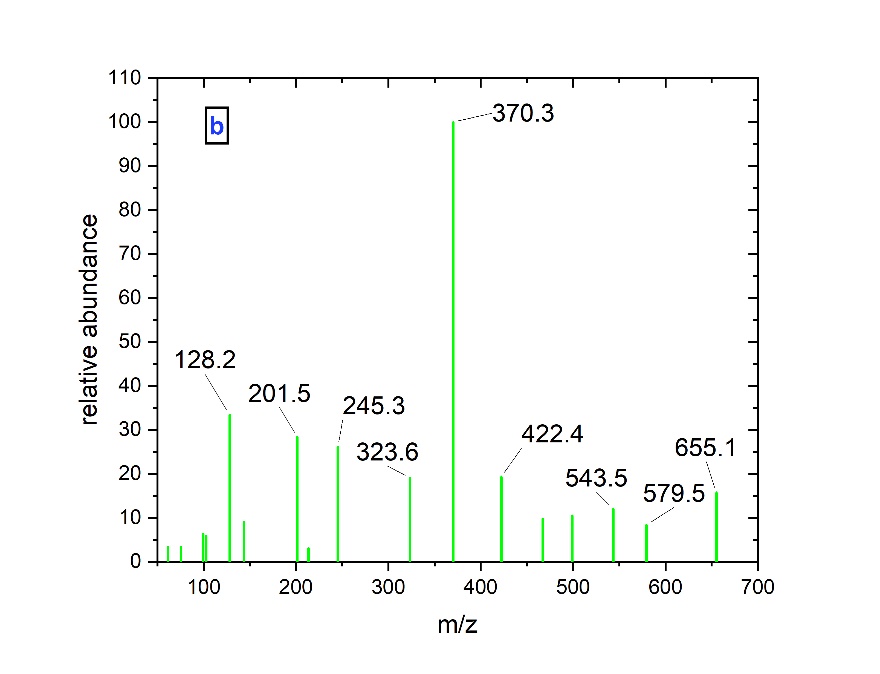


**Supplementary Figure 3.** Mass spectra of (a) Schiff base ligand and (b) Cu(II) complex


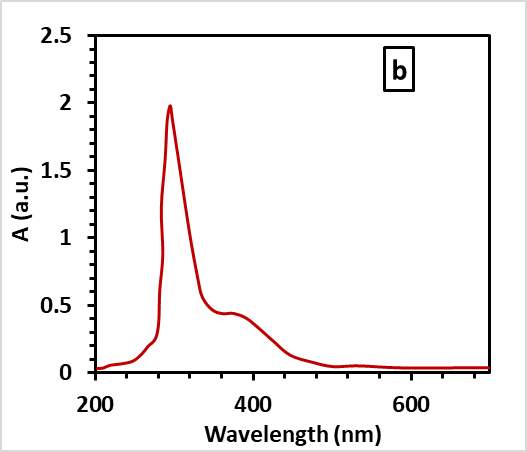

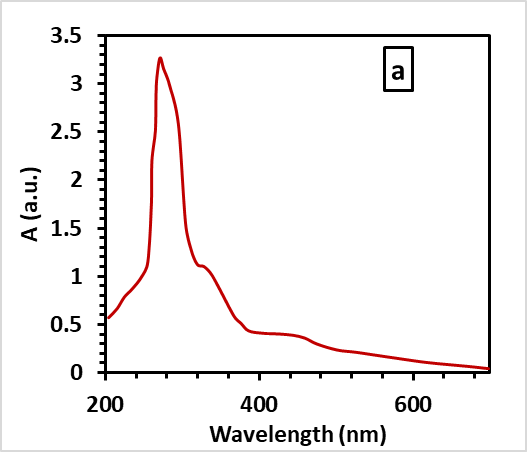


**Supplementary Figure 4.** UV-Vis spectra of (a) Ligand and (b) Cu(II) complex

**Biological Activity of Schiff base ligand and its Cu(II) complex:**

**Antimicrobial activity**

A filter paper disk (5 mm) was transferred into 250 Ml flasks containing 20 mL of working volume of tested solution (100 g/mL). All flasks were autoclaved for 20 min at 121 ºC. LB agar media surfaces were inoculated with four investigated bacteria (Gram positive and Gram negative) and two fungi organisms, then, transferred to a saturated disk with a tested solution in the center of Petri dish (agar plates). Finally, all these Petri dishes were incubated at 25 ºC for 48 h where clear or inhibition zones were detected around each disk. Control flask of the experiment was designed to perform under the same condition described previously for each microorganism but with N,N-dimethylformamide solution only and by subtracting the diameter of inhibition zone resulting with N,N-dimethylformamide from that obtained in each case, so antibacterial activity could be calculated ^1^. All experiments were performed as triplicate and data plotted were the mean value.

**Solutions for anticancer study**

The medium was used for culturing and maintenance of the human breast cell line. The medium was supplied in a powder form. It was prepared as follows: 10.4 g medium was weighed, mixed with 2 g sodium bicarbonate (Sigma Chemical Co., St. Louis, Mo, USA), completed to 1 L with distilled water and shacked carefully till complete dissolution. The medium was then sterilized by filtration in a Millipore bacterial filter (0.22 mL). The prepared medium was kept in a refrigerator (4 ºC) and checked at regular intervals for contamination. Before use the medium was warmed at 37 ºC in a water bath and the supplemented with penicillin/streptomycin and FBS.0.05% Isotonic trypan blue solution (Sigma Chemical Co., St. Louis, Mo, USA) was prepared in normal saline and was used for viability counting. 10% Fetal Bovine Serum (FBS) (heat inactivated at 56 ºC for 30 min), 100 units/mL Penicillin and 2 mg/mL Streptomycin were supplied from Sigma Chemical Co., St. Louis, Mo, USA and were used for the supplementation of RPMI-1640 medium prior to use. 0.025% (w/v) Trypsin (Sigma Chemical Co., St. Louis, Mo, USA) was used for the harvesting of cells. 1% (v/v) Acetic acid (Sigma Chemical Co., St. Louis, Mo, USA) was used for dissolving the unbound SRB dye. 0.4% Sulphorhodamine -B (SRB) (Sigma Chemical Co., St. Louis, Mo, USA) dissolved in 1% acetic acid was used as a protein dye. A stock solution of trichloroacetic acid (TCA, 50%, Sigma Chemical Co., St. Louis, Mo, USA) was prepared and stored. 50 mL of the stock was added to 200 ll RPMI-1640 medium/well to yield a final concentration of 10% used for protein precipitation. 100% Isopropanol and 70% ethanol were used. Tris base 10 mM (pH 10.5) was used for SRB dye solubilization. 121.1 g of tris base was dissolved in 1000 mL of distilled water and pH was adjusted by HCl (2 M).

**Anticancer activity**

Potential cytotoxicity of the compounds was tested using the method of Skehan *et al.* ^2^. Cells were plated in 96-multiwell plate (10^4^ cells/well) for 24 h before treatment with the compounds to allow attachment of cell to the wall of the plate. Different concentrations of the compounds under investigation (0, 5, 12.5, 25, 50 and 100 mg/mL) were added to the cell monolayer triplicate wells were prepared for each individual dose. The monolayer cells were incubated with the compounds for 48 h at 37 ºC and in 5% CO_2_ atmosphere. After 48 h, cells were fixed, washed and stained with SRB stain. Excess stain was washed with acetic acid and attached stain was recovered with tris–EDTA buffer. The optical density (O.D.) of each well was measured spectrophotometrically at 564 nm with an ELIZA microplate reader and the mean background absorbance was automatically subtracted and mean values of each compound concentration was calculated. The relation between surviving fraction and drug concentration is plotted to get the survival curve of breast tumor cell line for each compound. The equation used for calculating the percentage of cell survival was followed as previously described in many researches ^3,4^.

**References**

1. Sayed, F. N., Mahmoud, W. H., Omar, M. M. & Mohamed, G. G. Theoretical studies of new Schiff base ligand derived from 1,3-diaminopropane and 2-acetyl ferrocene and studying some applications of its metal complexes. *Appl Organomet Chem* **33**, (2019).

2. Skehan, P. *et al.* New Colorimetric Cytotoxicity Assay for Anticancer-Drug Screening. *JNCI Journal of the National Cancer Institute* **82**, 1107–1112 (1990).

3. Mahmoud, N. F., Abbas, A. A. & Mohamed, G. G. Synthesis, characterization, antimicrobial, and MOE evaluation of nano 1,2,4-triazole-based Schiff base ligand with some d-block metal ions. *Appl Organomet Chem* **35**, e6219 (2021).

4. Deghadi, R. G., Ahmed E., E., Ashraf M., A. & and Mohamed, G. G. Can One Novel Series of Transition Metal Complexes of Oxy-dianiline Schiff Base Afford Advances in Both Biological Inorganic Chemistry and Materials Science? *Comments on Inorganic Chemistry* **42**, 1–46 (2022).
